# The Clinical and Molecular Characterization of Gastric Cancer Patients in Qinghai-Tibetan Plateau

**DOI:** 10.3389/fonc.2020.01033

**Published:** 2020-06-30

**Authors:** Guanghong Rong, Yongxia Zhang, Yingcai Ma, Shilong Chen, Yongcui Wang

**Affiliations:** ^1^Department of Gastroenterology, Qinghai Provincial People's Hospital, Xining, China; ^2^Department of Gynecology, Qinghai Provincial People's Hospital, Xining, China; ^3^Key Laboratory of Adaptation and Evolution of Plateau Biota, Northwest Institute of Plateau Biology, Chinese Academy of Sciences, Xining, China; ^4^Institute of Sanjiangyuan National Park, Chinese Academy of Sciences, Xining, China; ^5^Qinghai Provincial Key Laboratory of Crop Molecular Breeding, Northwest Institute of Plateau Biology, Chinese Academy of Sciences, Xining, China

**Keywords:** gastric cancer, qinghai-tibetan plateau, clinical characteristics, molecular characteristics, the mechanism of cancer, cancer targeted therapeutics

## Abstract

Gastric cancer was the fifth most common malignancy and the third deadliest cancer (738,000 deaths in 2018) in the world. The analysis of its molecular characteristics has been complicated by histological and intratumor heterogeneity. Furthermore, the previous studies indicate that the incidence of gastric cancer shows wide geographical variation. As the largest and highest region in China, Qinghai-Tibetan Plateau (QTP) is one of the important global biodiversity hotspots. Here, to better understand the mechanism of gastric cancer and offer the targeted therapeutic strategies specially designed for patients in QTP, we collect tumor and blood samples from 30 primary gastric adenocarcinoma cancer patients at Qinghai Provincial People's Hospital. We discuss the clinical and molecular characteristics for these patients that have been ascribed to the unique features in this place, including high altitude (the average height above sea level is around 4,000 m), multi-ethnic groups, and the specific ways of life or habits (such as eating too much beef and mutton, have alcohol and cigarette problem, et al.). By comparing with the western gastric cancer patients collected from TCGA data portal, some unique characteristics for patients in QTP are suggested. They include high incidence in younger people, most of tumor are located in body, most of SNP are detected in chromosome 7, and the very different molecular atlas in minor ethnic groups and Han Chinese. These characteristics will provide the unprecedented opportunity to increase the efficacy for diagnosis and prognosis of gastric cancer in QTP. Furthermore, to suggest the targeted therapeutics specially designed for these 30 patients, an adapted kernel-based learning model and a compilation of pharmacogenomics data of 462 patient-derived tumor cells (PDCs) that illustrate the diverse genetic and molecular backgrounds of cancer patients, were introduced. In conclusion, our study offers a big opportunity to better understand the mechanism of gastric cancer in QTP and guide the optimal patient-tailored therapy for them.

## Introduction

Although it is steadily declining in incidence, cancer of the stomach (also known as gastric cancer) remains one of the most common and deadly malignancies in the world (1, 2). According to GLOBOCAN 2018 data, gastric cancer is the fifth most common malignancy and the third deadliest cancer (738,000 deaths annually), following only lung and colorectal cancer in overall mortality. Based on the reports from cancer.net in 2020, the 5-years survival rate is around 69%, if the cancer is diagnosed and treated before it has spread outside the stomach, will drop to 31%, if the cancer has spread to surrounding tissues or organs and/or the regional lymph nodes, and is 5%, if the cancer has spread to a distant part of the body.

Gastric cancer incidence and mortality were used to be related with Helicobacter pylori (H. pylori) infection. Recently, strides in preventing and treating H. pylori infection have already made the incidence declining. However, gastric cancer remains the fifth highest incidence among cancers, with 5.7% of all new cases attributable to the disease (3). A better understanding of the mechanism and risk factors of the disease can enable earlier diagnosis and thus lead to greater survival. Dietary modification, smoking cessation, and exercise hold promise in preventing gastric cancer. Furthermore, the previous studies suggested that the incidence of gastric cancer shows wide geographical variation (3, 4). More than 50% of the new cases occur in developing countries. East Asia (China and Japan), Eastern Europe, Central and South America are area with high occurrence, and Southern Asia, North and East Africa, North America, Australia, and New Zealand are area with low occurrence (4).

As the world's largest developing country with divergent geographical variation, China is characterized by large disparities in health care. Qinghai-Tibetan Plateau (QTP), the largest and highest region in China, is one of the important global biodiversity hotspots. QTP has a complex geological history, and it is a common understanding that the central plateau uplifted first and formed the ‘proto-QTP' as early as 40 Mya, followed by outward extensions in early Miocene (5–7). The late Miocene, 8–10 Mya, all the mountains surrounding QTP had reached steady elevation (8–11). Qinghai province is in northeast QTP. The average 4,000-m height above the sea level lead to the unique climate features there, including average temperature of 21 and −14 degrees centigrade in summer and winter, respectively, low humidity et al. People living there have their special dietary habit (such as eating too much beef and mutton), and always have alcohol and cigarette problem. All these features would result in unique characteristics of gastric cancer there, and should follow up the specific treatment strategy. In addition, according to the reports from Qinghai Provincial People's Hospital, the incidence of gastric cancer was the highest (640 new cases in 2019), followed by liver (427 new cases in 2019), lung (303 new cases in 2019), and colon cancer (237 new cases in 2019). Thus, it is urgent to analysis of its molecular and clinical characteristics for better understanding the mechanism of disease and guiding the optimal patient-tailored therapy.

Here, for better understanding the mechanism of gastric cancer and offer the targeted therapeutic strategies specially designed for patients in QTP, we collect tumor and blood sample from 30 primary gastric adenocarcinoma cancer patients at Qinghai Provincial People's Hospital, and discuss their unique clinical and molecular characteristics that have been ascribed to the unique features in this area. By comparing with the western gastric cancer patients collected from TCGA data portal (12), some unique characteristics for patients in QTP are suggested. They include high incidence in younger people, most of tumor are located in body, most of SNP are in chromosome 7, and the very different driver genes from that in western patients. The further analysis on driver genes indicates the different molecular atlas in minor ethnic groups and Han Chinese. Using an adapted kernel-based learning model and a compilation of pharmacogenomics data of 462 patient-derived tumor cells (PDCs) that illustrate the diverse genetic and molecular backgrounds of cancer patients (13), the specific targeted therapeutics for these 30 patients are suggested.

## Materials and Methods

### Tumor Specimens and Blood Sample

The tumor specimens and matched germline DNA samples (blood samples) with corresponding clinical records were obtained from patients undergoing surgery or gastroscopy with polypectomy at Qinghai Provincial People's Hospital. This work was performed in compliance with all relevant ethical regulations for research using human specimens. The fresh frozen tissue and blood samples were delivered to sequencing companies (ANOROAD GENOME and Sangon Biotech, Table S1) to capture the exonic DNA fragments and perform the whole exome sequencing. All patients here were acknowledged and signed the agreement with the hospital.

### The Pharmacogenomics Data that Used for Training Drug Response Learning Mode

The data used to build the drug response learning model came from the drug response data on the patient-derived cancer cells, which illustrate the diverse genetic and molecular backgrounds of cancer patients. It includes pharmacological landscapes of 462 patient-derived cancer cells across 60 small molecules, together with genomic and transcriptomic profiling in 385 of these tumors (13). The somatic mutations for 91 genes were used as the cancer genomics and the compound chemical structure was used to represent drug.

### TCGA Gastric Adenocarcinoma Cancer Patients and TCGA Drug Response Data

The clinical information for gastric adenocarcinoma cancer patients in TCGA data portal was collected (12). These information was used to compare with our plateau patients. The molecular properties for cancer patients, including somatic mutation and mRNA expression were collected from TCGA data portal to evaluate the prediction model in revealing the effective drugs for cancer patients. The clinical drug responses to TCGA cancer patients came from Ding's work (14). As did in (14), we removed possible combination therapy and chemotherapy earlier than surgery, and kept 2,182 patient-drug pairs (including 1,029 pan-cancer patients and 130 clinical drug) for evaluation.

### Cancer Cell Line Pharmacogenomics

To illustrate the advantage of using drug sensitivities in patient-derived cancer cells in prediction of the effective drugs for cancer patients, the well-established cancer genomic resources CTPR (15) was introduced. CTRP links genetic, lineage, and other cellular features of cancer cell lines to small-molecule sensitivity, and extended 24 compounds in Cancer Cell Line Encyclopedia (CCLE) (16) to ‘Informer Set' compounds (including 481 small molecule probes and drugs). Specifically, the mutation status for 25 oncogenes across 486 cancer cells, and mRNA expressions for 54,675 mRNAs across 127 cancer cells were obtained from CCLE. The dose-response curve (AUCDR) for 545 drugs across 887 cancer cell lines were extracted from CTPR.

### Whole Exome Sequencing and SNP Calling

The Illumina HiSeq 2000 instrument was used for sequencing and generated 2 × 150 base paired-end reads. FASTQ files were aligned to the human genome assembly (hg38) via Burrows–Wheeler Aligner (BWA) (17). Before further analysis, the initially aligned BAM files were subjected to preprocessing that sorted, removed duplicated reads, locally realigned reads around potential small indels, and recalibrated base quality scores using SAMtools (18), and Picard (https://broadinstitute.github.io/picard/). The single Nucleotide Polymorphsim, SNP was then detected through the Genome Analysis ToolKit (GATK) (19). After removing the duplicated and low quality SNPs, the remaining SNPs were annotated via ANNOVAR (20).

### Genome Coverage, Somatic Mutation, and Gene Fusion Analysis

The genomecov function under bedtools package (21) was used to calculate the coverage per-base based on preprocessed BAM files. Using the human genome (hg38) annotation file, the coverage at gene level was obtained. The somatic mutation was obtained by using germline SNPs from blood samples as a reference. The FusionMap (22) was introduced to perform the gene fusion analysis based on FASTQ files.

### Identification of Cancer Driver Gene

The MaxMIF (23), which outperformed the existing state-of-the-art methods (including MUFFINN (24), MuttSig2 (25), MutSigCV (26), et al.) on TCGA pan-cancer datasets, was introduced to distinguish the cancer driver genes from the passenger genes. MaxMIF integrated the somatic mutation data and molecular interaction data by a maximal mutational impact function. The protein-protein interaction (PPI) network deposited in HumanNet (27) was introduced to represent the molecular interactions.

### An Adapted Kernel-Based Learning Model for Prediction of the Effective Clinical Drugs

We applied our previously adapted kernel-based learning model to predict the effective clinical drugs for cancer patients (28). To simultaneously consider all available drugs across all cancer cell lines, we introduced the bipartite graph framework under the assumption that drugs with similar chemical properties should have similar treatment outcomes (28). To overcome the difficulty of heterogeneous data integration, we adapted a kernel method to construct similarity matrix based on different types of feature. Specifically, the cancer genomic data (such as mutation, expression, et al.) and chemical properties were applied to construct kernel-based similarity matrices between cancer cell lines and anticancer drugs. Based on experimentally tested drug sensitivities, a bipartite graph was constructed to represent the relationships between cancer cells and anticancer drugs, where the edges were labeled by the digitalized drug sensitivities. The three classification models, random forest (RF), support vector machine (SVM), and deep learning network (DN) were then utilized on these kernel-based similarity matrices to predict the effective clinical drugs for cancer patients. The RF, SVM and DN model were implemented via “randomForest” R package with default parameters, LibSVM (29) in “e1071” R package with RBF kernel function, and the “h2o” R package with default parameters, respectively. The penalty parameter and the RBF kernel parameter were optimized by the grid search approach with 5-fold cross-validation. The area under the ROC curve (AUC) (30) was used as the evaluation criteria to assess the performance of classification model.

## Results

### Clinical Characteristics

**Figure 1:**
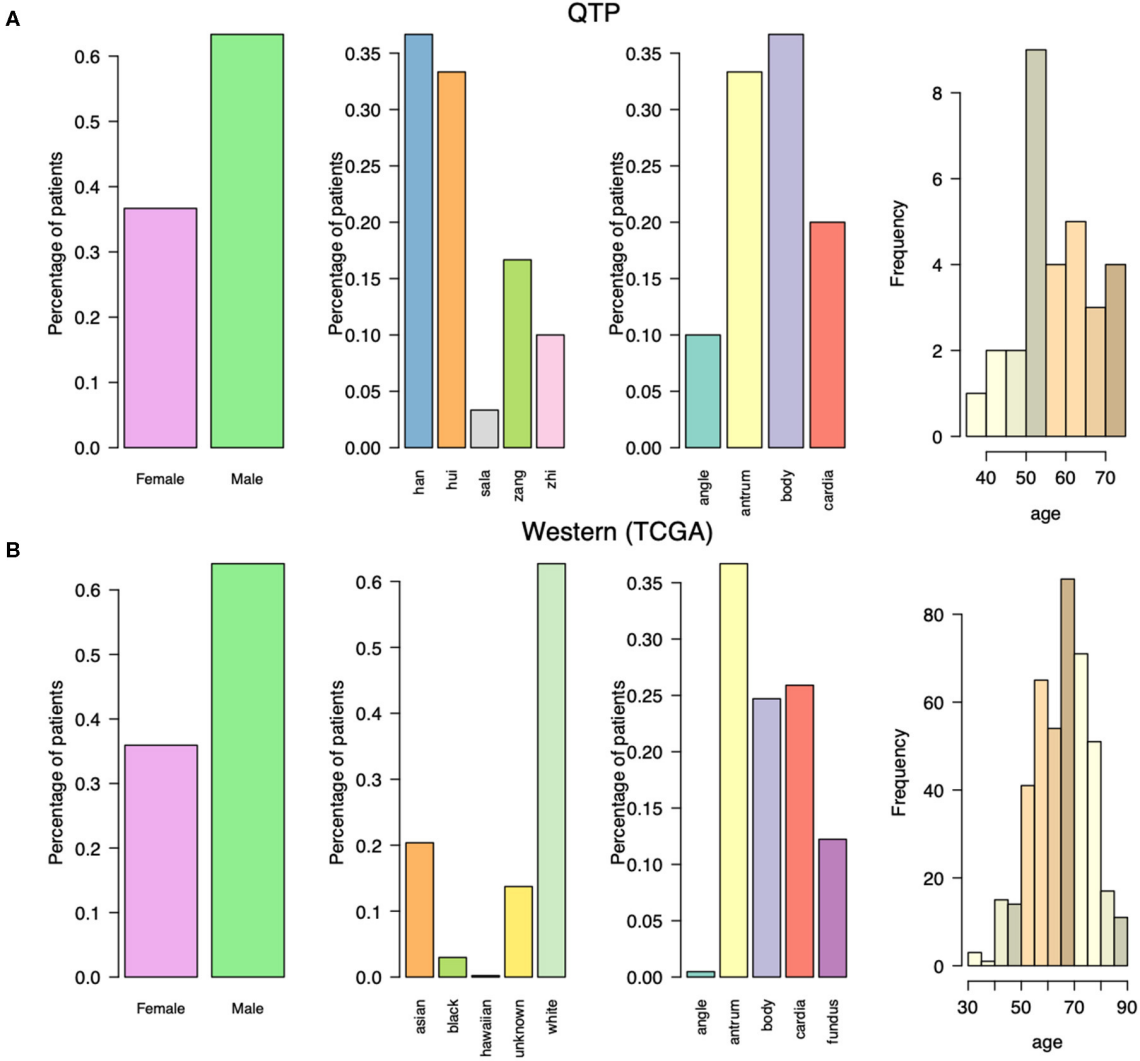
The clinical characteristics for gastric adenocarcinoma patients collected from Qinghai Provincial People's Hospital and TCGA data portal. **(A)** The gender, ethnic group, tumor location, and initial diagnosis age for gastric adenocarcinoma patients collected from Qinghai Provincial People's Hospital. **(B)** The gender, ethnic group, tumor location, and initial diagnosis age for gastric adenocarcinoma patients collected from TCGA data portal.

We obtained tumor tissue (fresh frozen) from 30 primary gastric adenocarcinoma cancer patients not treated with prior chemotherapy or radiotherapy (Table S1). We collected some clinical information for these cancer patients (Figure 1A), including the initial diagnosis age, the gender, the location of primary tumor tissue, and the ethnic group information, and compared them with western gastric adenocarcinoma patients which collected from TCGA (Figure 1B). Both western and plateau patients have higher incidence in male patients. The most tumors of plateau patients are located in body, while the antrum tumors are the most for western patients. Comparing with the western patients in terms of initial diagnosis age, plateau patients are younger. They are around fifty, and western patients are around sixty. The differences in clinical properties indicate the unique molecular characteristics for these plateau patients that differ with western patients, and should follow by the specific treatment strategy.

### Molecular Characteristics

**Figure 2:**
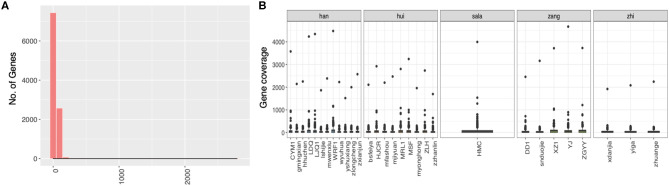
The distribution of genome coverage at gene level in gastric adenocarcinoma patients collected from Qinghai Provincial People's Hospital. **(A)** The distribution of genome coverage at gene level. **(B)** The distribution of genome coverage at gene level along the patients.

**Figure 3:**
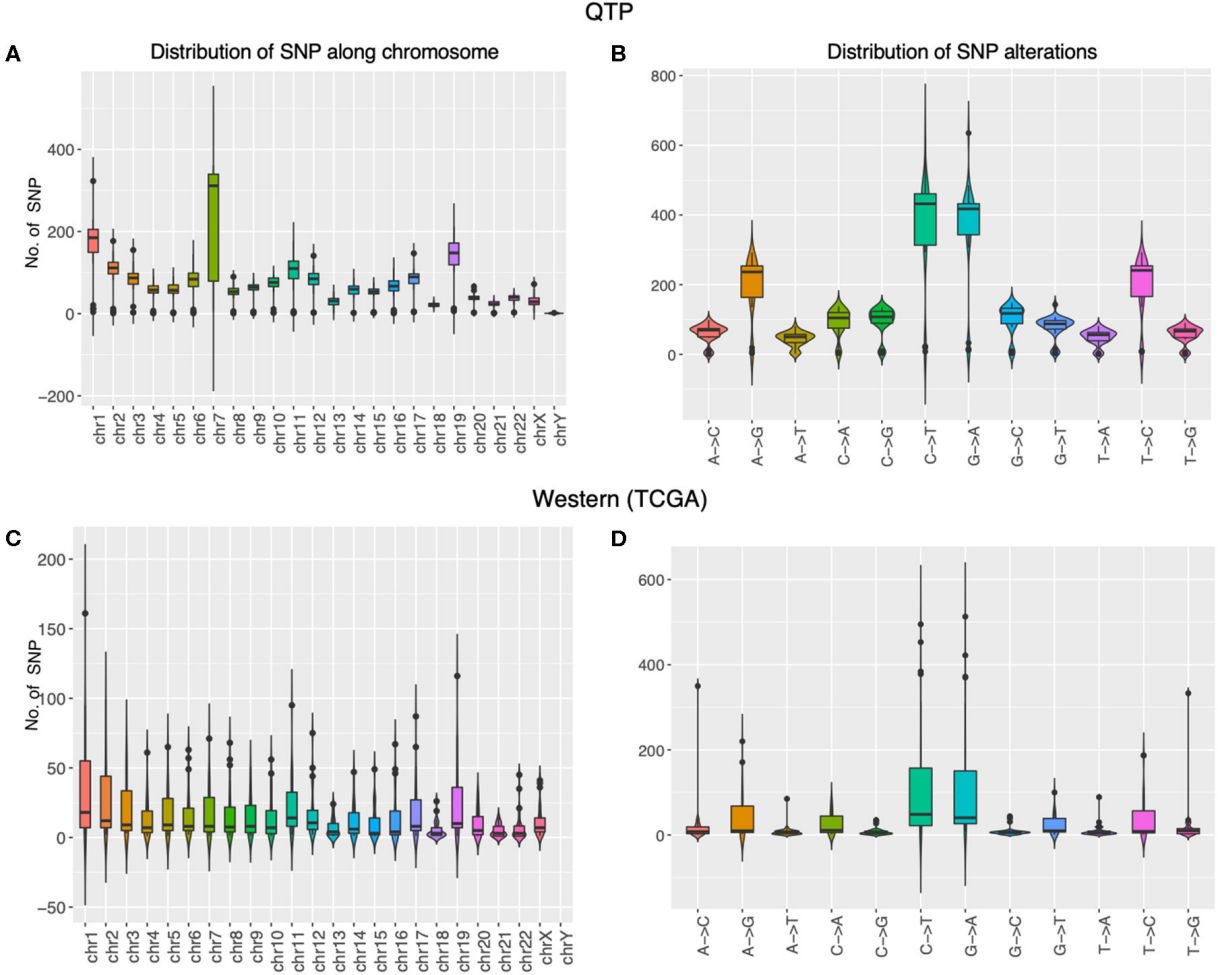
The molecular properties of gastric adenocarcinoma patients collected from Qinghai Provincial People’s Hospital and TCGA data portal. The panels **(A,B)** display the distribution of SNP along the chromosome and the distribution of SNP alternation types in gastric adenocarcinoma patients collected from Qinghai Provincial People’s Hospital, and the panels **(C,D)** display the distribution of SNP along the chromosome and the distribution of SNP alternation types in gastric adenocarcinoma patients collected from TCGA data portal.

We first check the sequencing depth at gene level via counting the genome coverage along the gene loci. Most of genes have depth around a hundred (Figure 2A) and gene coverage shows the different distribution for different ethnic group of patients (Figure 2B). Then we discuss the SNP distribution along the chromosome and compare them with western patients. The SNP distribution along the chromosome is more stable in western patients than that in plateau patients (Figures 3A,C), and the compared with other chromosome, more SNPs were detected in chromosome 7 for plateau patients. According to the database of COSMIC (31), the world's largest and most comprehensive resource for exploring the somatic mutations in human cancer, chromosome 7 consists of lots of well-known cancer-related somatic mutations, including EGFR, BRAF, CDK6, MET, T1F1, and so on. The copy number variation in chromosome 7 was also related with cancer (32). These results together indicate the important region was detected and the biomarkers for further diagnosis and treatment can be found in chromosome 7. As for SNP alteration type, both western and plateau patients show the majority of C->T and G->A alteration types, and compared with western patients, the variations in each alteration type are bigger in plateau patients than that in western patients (Figures 3B,D).

**Figure 4:**
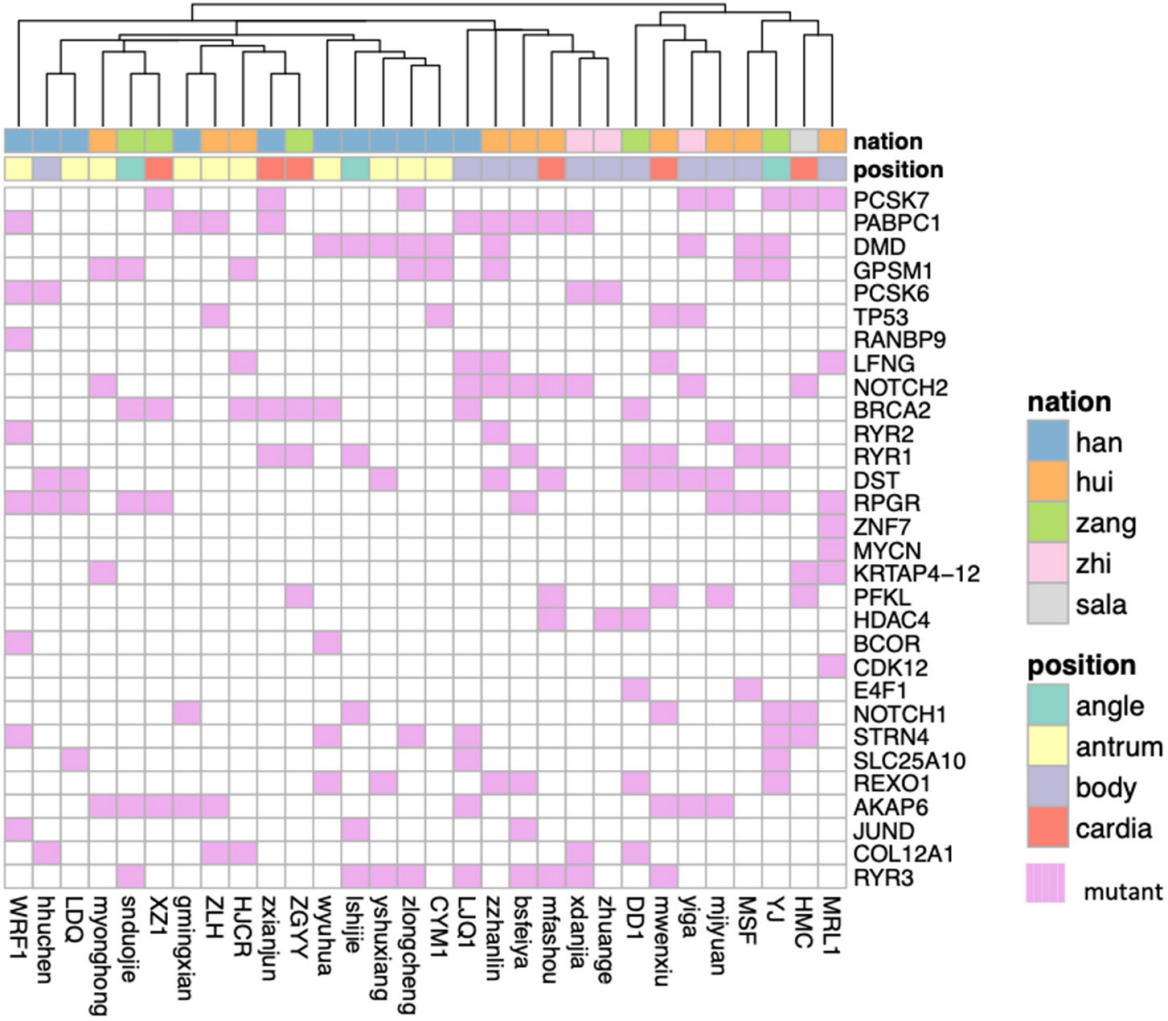
The heatmap shows the cancer driver genes obtained based on MaxMiF in gastric adenocarcinoma patients collected from Qinghai Provincial People's Hospital. The ethnic group and the tumor location are shown with different colors.

Using MaxMIF on above somatic mutation data and PPIs from humanNet (27), the first 30 genes with highest MaxMIF scores were selected as the cancer driver genes (Figure 4). We also performed MaxMIF on western patients, and selected the top 30 genes as the cancer driver genes (Figure S1). Only 5 genes (TP53, RYR2, RYR1, COL12A1, DST) were presented in cancer driver gene list for both plateau and western patients, meaning the molecular differences in plateau patients and western patients. From Figure 4, the two different groups of patients can be found. One is for minor ethnic groups, and the other is Han Chinese and a few of minor ethnic groups of patients, suggesting the difference molecular properties of Han Chinese with minor ethnic groups. In addition, the gene fusion analysis shows in-frame gene fusion of the number 2 exon of KRTAP10-7 and number 1 exon of KRTAP10-6, and number 28 exon of IPO4 and number 21 exon of DNHD1 (Table S2). It also identified the frame-shift gene fusion of the number 1 exon of AKR7A3 and number 11 exon of AKR7L. However, TCGA Research Network only reported in-frame gene fusion of CLDN18 and ARHGAP in western patients (12).

In sum, the unique clinical and molecular characteristics for plateau patients were detected, meaning that there should have some treatment strategies that are specifically designed for them.

### Prediction of Effective Clinical Therapies in Plateau Patients

The big challenge in cancer patient treatment is the intratumoral heterogeneity (ITH), a phenomenon observed in a number of aggressive cancers which consist of heterogeneous cell populations with a wide range of morphologies, genotypes and phenotypes. Increasing evidences suggested that ITH plays an important role in drug resistance (33–35). Thus, to detect the effective clinical drugs for these plateau patients that have different clinical and molecular characteristics with western patients, we trained our previously adapted kernel-based learning model (28) on pharmacogenomics generated from patient-derived cancer cells (gliomaScan) that could illustrate the phenomenon of ITH in terms of drug response (13). To evaluate the prediction model, we introduced the clinical drug responses on TCGA cancer patients as the independent testing data. Compared with using cancer genomics generated from cancer cell lines (CTRP) as the training data, trained on cancer genomics generated from patient-derived cancer cells obtained more accurate clinical responses for TCGA patients, no matter using which types of machine-learning algorithms (Figure S2).

**Figure 5:**
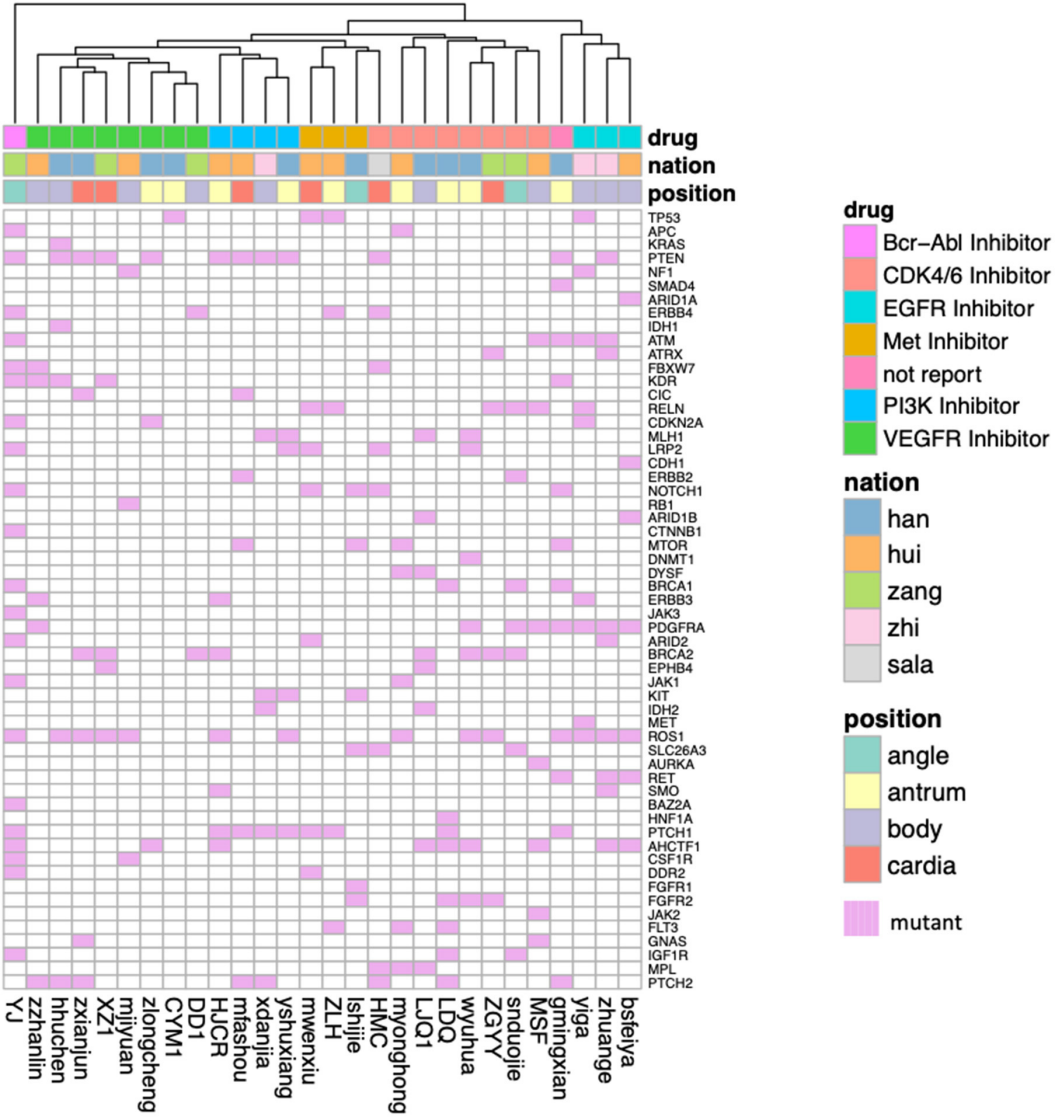
The predicted effective clinical therapy for gastric adenocarcinoma patients collected from Qinghai Provincial People's Hospital. The patients were grouped by the types of kinase inhibitors, and the oncogenes showed here are those somatic mutations in gliomaSCAN. The ethnic group and the tumor location are shown with different colors.

Then we applied above prediction model to predict the effective drugs for our plateau patients. The predictions are well-grouped as the types of kinase inhibitors (Figure 5). Not as the clusters obtained on cancer driver genes, there are no clear classification among Han Chinese and minor ethnic groups of patients, and they will share some target therapies. Among all 30 patients, we found one special minor ethnic group patient (Tibetan people, zang) that was predicted to be treated by Bcr-Abl inhibitor, Bosutinib. Bosutinib is approved to treat chronic myelogenous leukemia (CML) according to NCI reports, and is usually given after other treatments have failed (36). It is not related with gastric cancer yet. Therefore, it might be the good option for that patient if other treatments failed.

## Discussion and Conclusion

Qinghai province is in northeast QTP with average 4,000-m height above sea level. This place has its unique climate features, including low temperature (average 21 degrees of centigrade in summer) and low humidity. Han Chinese and lots of minor ethnic groups (such as hui, zang (Tibetan people), zhi, sala, and so on) live here, and they like to eat mutton and beef. In addition, people living here always have alcohol and cigarette problem. The gastric cancer, which are related with dietary and lifestyles, is the most common cancer types in Qinnghai province according to the statistical report in Qinghai Provincial People's Hospital in 2019. Here, to better understand the molecular mechanism of gastric cancer in QTP, and predict the most effective targeted therapeutics for patients there, we collected the tumor and blood samples from primary gastric adenocarcinoma cancer patients at Qinghai Provincial People's Hospital, and discussed the clinical and molecular characteristics for those patients. By comparing with the western patients collected form TCGA, we found some unique characteristics for our plateau patients, including younger initial diagnosis age, most of tumors located in body of stomach, only share 5 driver genes with TCGA western patients, have unique gene fusions, et al. Importantly, the cancer driver gene analysis indicates the differences in Han Chinese and minor ethnic groups. For instance, only one of 12 Han Chinese have NOTCH2 mutant, and 7 of 18 minor ethnic people have that gene mutant. It is worthy for further validation by wet-lab *in vivo* and *in vitro* in the future work. In addition, drug response prediction mode based on pharmacogenomics from patient-derived cancer cells suggests the effective targeted therapies, which specifically designed for these plateau patients. The prediction results could be evaluated by following up tracking reports. In future, we will collect more patients and their follow-up treatment information to validate the predictions here. In addition, the tumor bio-samples were returned to our lab after sequencing. Thus, we will perform the drug-screening assay on these samples to further validat our predicted therapies.

There are around 5,000,000 people living in Qinghai province, and based on the recent statistic reports, only around 1,000,000 people live in Xining. The sample and follow-up treatment information collections are quite challenge here. It took us 2 years to collect these 30 patients. In future, we will collect much more patients with the treatment reports to further show the unique characteristics of plateau patients and discuss their special treatment strategy.

## Data Availability Statement

The raw data supporting the conclusions of this article will be made available by the authors, without undue reservation, to any qualified researcher.

## Ethics Statement

The studies involving human participants were reviewed and approved by Qinghai Provincial People's Hospital. The patients/participants provided their written informed consent to participate in this study.

## Author Contributions

GR and YZ conceived the idea and supervised the study. YW designed and implemented the prediction model, and performed data analysis. GR and YZ analyzed the results and drafted the manuscript. SC edited the manuscript. All authors read and approved the final manuscript.

## Conflict of Interest

The authors declare that the research was conducted in the absence of any commercial or financial relationships that could be construed as a potential conflict of interest.
