# Supplementary material for: The Clinical and Molecular Characterization of Gastric Cancer Patients in Qinghai-Tibetan Plateau
Source: Front Oncol. 2020 Jun 30;10:1033. doi: 10.3389/fonc.2020.01033 (PMC7339979; doi:10.3389/fonc.2020.01033)

Supplementary Materials

The clinical and molecular characterization of gastric cancer patients in Qinghai-Tibetan Plateau

Guanghong Rong ^1#^, Yongxia Zhang ^2#^, Yingcai Ma^1^, Shilong Chen^3,4^, Yongcui Wang^4,5*^

^1^ Department of Gastroenterology, Qinghai Provincial People’s Hospital, Xining, Qinghai, 810007, China

^2^ Department of Gynecology, Qinghai Provincial People’s Hospital, Xining, Qinghai, 810007, China

^3^ Key Laboratory of Adaptation and Evolution of Plateau Biota, Northwest Institute of Plateau Biology, Chinese Academy of Sciences, Xining, Qinghai, 810008, China

^4^ Institute of Sanjiangyuan National Park, Chinese Academy of Sciences, Xining, 810008, China

^5^ Qinghai Provincial Key Laboratory of Crop Molecular Breeding, Northwest Institute of Plateau Biology, Chinese Academy of Sciences, Xining, 810008, China

*** Correspondence:**Corresponding Author
[ycwang@nwipb.cas.cn](mailto:ycwang@nwipb.cas.cn)

^#^ These authors contributed equally to this work as first authors.

Legend

Table S1.

The summary of tumor and blood samples in this study.

Table S2.

The detail of bio-samples in this study.

Table S3.

The details of fusion genes obtained by FusionMap.

Figure S1

The heatmap shows the top 30 driver genes in TCGA gastric patients. The ethnic group and tumor location were shown with different colors.

Figure S2

The prediction performance (AUC) on prediction of the clinical drug response in TCGA patients by using pharmacogenomics generated from cancer cell lines and patient-derived cancer cells.

Table S1

| company | ANOROAD GENOME | Sangon Biotech |
| --- | --- | --- |
| platform | Illumina HiSeq | Illumina HiSeq |
| No. of bio-sample collected | 17 tissue | 14 tissue, 9 matched blood sample |
| No. of bio-sample sequenced | 17 tissue | 13 tissue, 4 matched blood sample |

Table S2

| **sample** | **type** | **gender** | **ID** | **label** | **nationality** | **age** |
| --- | --- | --- | --- | --- | --- | --- |
| zxianjun | tissue | Male | Case1 | cardia | han | 51 |
| zlongcheng | tissue | Male | Case2 | antrum | han | 56 |
| xdanjia | tissue | Male | Case3 | body | zhi | 63 |
| lshijie | tissue | Male | Case4 | angle | han | 73 |
| myonghong | tissue | Male | Case5 | antrum | hui | 62 |
| snduojie | tissue | Male | Case6 | angle | zang | 43 |
| yiga | tissue | Male | Case7 | body | zhi | 63 |
| zhuange | tissue | Female | Case9 | body | zhi | 71 |
| mfashou | tissue | Male | Case10 | cardia | hui | 52 |
| mjiyuan | tissue | Male | Case12 | body | hui | 53 |
| mwenxiu | tissue | Male | Case13 | cardia | han | 74 |
| hhuchen | tissue | Male | Case14 | body | han | 54 |
| gmingxian | tissue | Male | Case15 | antrum | han | 54 |
| wyuhua | tissue | Female | Case16 | antrum | han | 39 |
| bsfeiya | tissue | Female | Case17 | body | hui | 57 |
| zzhanlin | tissue | Male | Case18 | body | hui | 64 |
| yshuxiang | tissue | Female | Case19 | antrum | han | 70 |
| ZLH | tissue | Female | case18 | antrum | hui | 51 |
| ZGYY | tissue | Female | case17 | cardia | zang | 47 |
| HJCR | tissue | Male | case1 | antrum | hui | 69 |
| MRL1 | tissue | Female | case5 | body | hui | 57 |
| MRL2 | blood | Female | case6 | body | hui | 57 |
| CYM1 | tissue | Female | case22 | antrum | han | 46 |
| CYM2 | blood | Female | case23 | antrum | han | 46 |
| DD1 | tissue | Female | case24 | body | zang | 43 |
| DD2 | blood | Female | case25 | body | zang | 43 |
| LDQ | tissue | Male | case3 | antrum | han | 51 |
| XZ1 | tissue | Female | case15 | cardia | zang | 52 |
| LJQ1 | tissue | Male | case4 | body | han | 58 |
| HMC | tissue | Male | case2 | cardia | sala | 65 |
| YJ | tissue | Female | case16 | angle | zang | 72 |
| MSF | tissue | Male | case7 | body | hui | 69 |
| WYM1 | tissue | Female | case13 | colon | han | 54 |
| WYM2 | blood | Female | case14 | colon | han | 54 |
| WRF1 | tissue | Male | case11 | antrum | han | 55 |
| WRF2 | blood | Male | case12 | antrum | han | 55 |
| ZP22 | tissue | Female | case21 | normal | han | 58 |

Table S3

| platform | No. of patients with fusion gene reports | Fusion gene (exon number) | FrameShiftClass |
| --- | --- | --- | --- |
| ANOROAD GENOME | 5 (17) | KRTAP10-7(2)->KRTAP10-6 (1) | InFrame |
| Sangon Biotech | 7(13) | IPO4 (28)->DNHD1(21)  KRTAP10-7(2)->KRTAP10-6 (1)  AKR7A3 (1)->AKR7L (11) | InFrame  InFrame  FrameShift |

Figure S1.


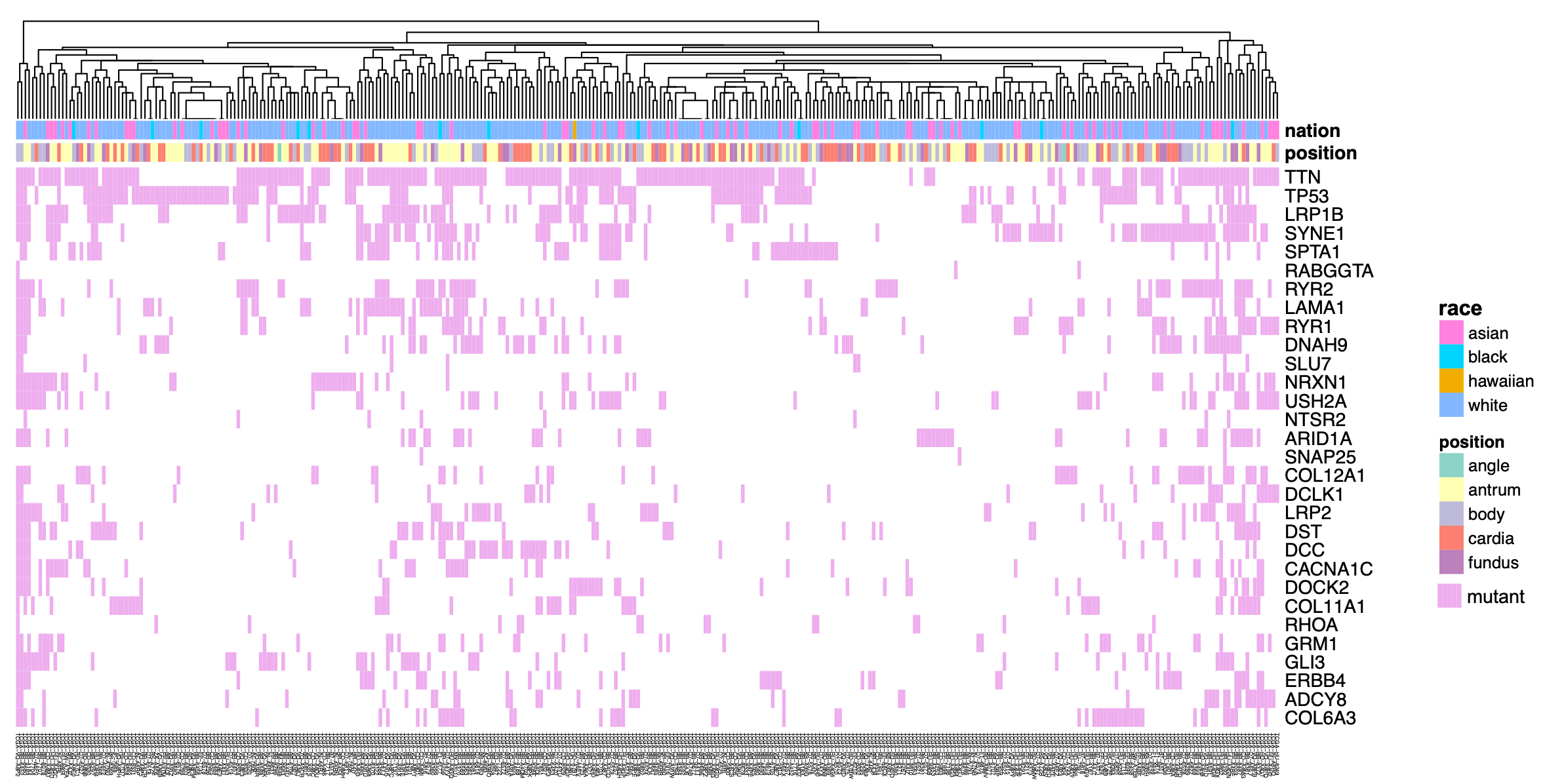


Figure S2.


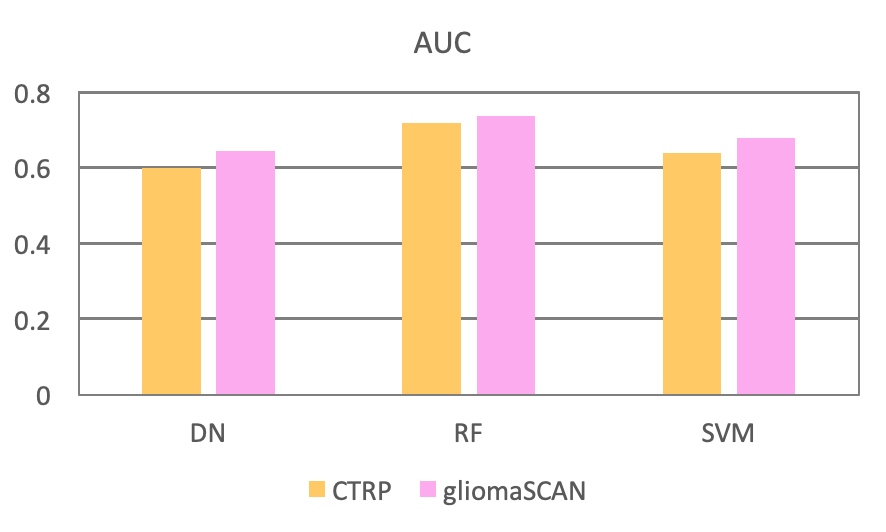

Supplement: Supplementary file 1 [file Data_Sheet_1.docx]
